# *Listeria cossartiae* sp. nov., *Listeria immobilis* sp. nov., *Listeria portnoyi* sp. nov. and *Listeria rustica* sp. nov., isolated from agricultural water and natural environments

**DOI:** 10.1099/ijsem.0.004795

**Published:** 2021-05-17

**Authors:** Catharine R. Carlin, Jingqiu Liao, Dan Weller, Xiaodong Guo, Renato Orsi, Martin Wiedmann

**Affiliations:** ^1^​ Department of Food Science, Cornell University, Ithaca, NY 14853, USA; ^2^​ Department of Microbiology, Cornell University, Ithaca, NY 14853, USA; ^†^​ Present address: Department of Systems Biology, Columbia University, New York, NY 10032, USA; ^‡^​ Present address: Department of Environmental and Forest Biology, SUNY College of Environmental Science and Forestry, Syracuse NY 13210, USA

**Keywords:** ANI, GTDB-Tk, *Listeria sensu stricto*, *Listeria sensu lato*, novel species

## Abstract

A total of 27 *Listeria* isolates that could not be classified to the species level were obtained from soil samples from different locations in the contiguous United States and an agricultural water sample from New York. Whole-genome sequence-based average nucleotide identity blast (ANIb) showed that the 27 isolates form five distinct clusters; for each cluster, all draft genomes showed ANI values of <95 % similarity to each other and any currently described *Listeria* species, indicating that each cluster represents a novel species. Of the five novel species, three cluster with the *Listeria sensu stricto* clade and two cluster with *sensu lato*. One of the novel *sensu stricto* species, designated *L. cossartiae* sp. nov., contains two subclusters with an average ANI similarity of 94.9%, which were designated as subspecies. The proposed three novel *sensu stricto* species (including two subspecies) are *Listeria farberi* sp. nov. (type strain FSL L7-0091^T^=CCUG 74668^T^=LMG 31917^T^; maximum ANI 91.9 % to *L. innocua *), *Listeria immobilis* sp. nov. (type strain FSL L7-1519^T^=CCUG 74666^T^=LMG 31920^T^; maximum ANI 87.4 % to *L. ivanovii* subsp. *londoniensis *) and *Listeria cossartiae* sp. nov. [subsp. *cossartiae* (type strain FSL L7-1447^T^=CCUG 74667^T^=LMG 31919^T^; maximum ANI 93.4 % to *L. marthii *) and subsp. *cayugensis* (type strain FSL L7-0993^T^=CCUG 74670^T^=LMG 31918^T^; maximum ANI 94.7 % to *L. marthii *). The two proposed novel *sensu lato* species are *Listeria portnoyi* sp. nov. (type strain FSL L7-1582^T^=CCUG 74671^T^=LMG 31921^T^; maximum ANI value of 88.9 % to *L. cornellensis* and 89.2 % to *L. newyorkensis *) and *Listeria rustica* sp. nov. (type strain FSL W9-0585^T^=CCUG 74665^T^=LMG 31922^T^; maximum ANI value of 88.7 % to *L. cornellensis* and 88.9 % to *L *. *newyorkensis *). *L. immobilis* is the first *sensu stricto* species isolated to date that is non-motile. All five of the novel species are non-haemolytic and negative for phosphatidylinositol-specific phospholipase C activity; the draft genomes lack the virulence genes found in *Listeria* pathogenicity island 1 (LIPI-1), and the internalin genes *inlA* and *inlB*, indicating that they are non-pathogenic.

As of 29 October 2020, there were 21 recognized species and six subspecies representing the genus *Listeria *, which can be divided into two distinct clades, *sensu stricto* (* L. monocytogenes* [1], *L. innocua* [2], *L. ivanovii* [3] including subsp. *ivanovii* and *londoniensis* [4], *L. seeligeri* [5], *L. marthii* [6], *L. welshimeri* [5]) and *sensu lato* (* L. grayi* [7] including subsp. *grayi* and *murrayi* [7]*, L. fleischmannii* [8] including subsp. *fleischmannii* and *coloradonensis* [9], *L. floridensis* [10], *L. aquatica* [10], *L. costaricensis* [11], *L. goaensis* [12], *L. thailandensis* [13], *L. valentina [14]*, *L. newyorkensis* [15], *L. cornellensis* [10], *L. rocourtiae* [16], *L. weihenstephanensis* [17], *L. grandensis* [10], *L. riparia* [10], *L. booriae* [15]). The latest *Listeria* species reported is *L. valentina *, a *sensu lato* species published on 5 October 2020. Over the past 10 years, 15 new species [11–14, 18] have been added to the genus *Listeria *, and all but one (* L. marthii* [6]) were added to the *sensu lato* clade. *L. marthii* was described in 2010 and the other five *sensu stricto* species were identified before 1985 [18]. In our analyses of soil and water samples, we identified 27 isolates that could not be classified to the species level based on *sigB* sequencing, a rapid method for initial *Listeria* characterization and speciation routinely used by our group [19]. Average nucleotide identity blast (ANIb) analysis of whole-genome sequencing data yielded five phylogenetic clusters, each cluster representing a novel species. Three of the novel species (*L. farberi*, *L. immobilis*, *L. cossartiae* subsp. *cossartiae* and subsp. *cayugensis*) group into the *sensu stricto* clade, the clade of interest to public health as it contains recognized human and animal pathogens (i.e. *L. monocytogenes* and *L. ivanovii *) [20–23]. The two other novel species (*L. portnoyi* and *L. rustica*) group into the *sensu lato* clade.

## Bacterial strain collection and isolation

The isolates characterized here were obtained from two separate studies: one assessing the diversity and prevalence of *Listeria* in the soil in natural environments [24] and one evaluating foodborne pathogens isolated from agricultural water sources [25]. The soil and water samples collected in these studies yielded 27 isolates that were identified as *Listeria* but could not be classified to the species level. Of the 27 isolates, 26 were obtained from soil and one (FSL W9-0585^T^) from water. The specific geographical locations and GPS coordinates for all 27 isolates are provided in Table S1 (available in the online version of this article); isolates with the same coordinates originated from the same sample. The isolates collected from soil originated from samples collected in rural regions in eight US states. *L. cossartiae* subsp. *cayugensis* was isolated from two soil samples, one from North Carolina and the other from Georgia. *L. cossartiae* subsp. *cossartiae* was isolated from six soil samples, all collected in Alabama. *L. farberi* was isolated from five different soil samples collected in Texas (*n*=3) and Florida (*n*=2). *L. immobilis* was isolated from seven different soil samples collected in Montana (*n*=2), South Dakota (*n*=4), and Wyoming (*n*=1); one of the soil samples collected in South Dakota also yielded *L. portnoyi. L. rustica* was isolated from an agricultural water source in New York. Enrichment and isolation of *Listeria* was conducted as described in the FDA BAM Chapter 10 [26]. Briefly, 25 g or mL of soil or water were enriched in Buffered *Listeria* Enrichment Broth (BLEB; Becton Dickinson) with selective supplements (* Listeria* Selective Supplement, Oxoid) added after 4 h of incubation at 30 °C. BLEB enriched soil or water samples were streaked for isolation after 24 and 48 h of incubation onto modified Oxford *Listeria* selective agar (MOX; Becton Dickinson) and R&F *Listeria monocytogenes* Chromogenic Plating Medium (LMCPM; R&F Laboratories). MOX and LMCPM plates were incubated at 30 and 35 °C, respectively, for 48 h after which up to eight presumptive *Listeria* colonies were selected from both plate types and isolated onto BHI. Individual isolates were selected from BHI for initial characterization using a previously described protocol for PCR amplification and sequencing of the partial *sigB* gene [27]. All of the novel species described here could not be placed into any existing species based on *sigB* sequence data. Further genetic and phenotypic characterization were thus performed using pure cultures of all 27 isolates; cultures were stored at −80 °C in brain heart infusion (BHI; Becton Dickinson) broth supplemented with 15 % glycerol.

## Whole-genome sequencing and phylogenetic analysis

The 27 isolates that could not be assigned to a known *Listeria* species underwent whole-genome sequencing (WGS) to allow for further characterization and phylogenetic analyses. Genomic DNA was extracted using the QIAamp DNA MiniKit (Qiagen) per the manufacturer’s protocol for Gram-positive bacteria. The extracted DNA quality was assessed using OD_260_/OD_280_ and OD_260_/OD_230_ values obtained on the Nanodrop [28]; DNA concentration was determined using Qubit [29]. Library preparation was performed using the Nextra XT (Illumina) kits. Whole-genome sequencing was completed using either Illumina’s MiSeq (2×250 bp reads; *L. farberi* isolates FSL L7-0072, FSL L7-0083, FSL L7-0091^T^ and *L. rustica* FSL W9-0585^T^), HiSeq2500 (2×150 bp reads; all isolates representing *L. cossartiae* subsp. *cossartiae*, *L. cossartiae* subsp. *cayugensis*, *L. immobilis* and *L. portnoyi*) or NextSeq500 [2×150 bp reads; *L. farberi* isolates FSL L7-1693 and FSL L7-1699) platforms. Draft genomes were assembled as described by Kovac *et al*. [30]. Briefly, adapter sequences were trimmed using Trimmomatic version 0.39 [31]. Paired-end reads were assembled *de novo* using SPAdes version 3.13.1 with k-mer sizes of 21, 33, 55 and 77 for Illumina HiSeq and NextSeq, and 33, 55, 77, 99 and 127 for MiSeq [32]. Contigs <500 bp were removed, and assembly quality was checked using quast version 5.0.2 [33]. The quast output was reviewed to verify the draft genomes met the following quality criteria: (i) N50 >50 000; (ii) total number of contigs <300; and (iii) average coverage >30×. The G+C content and draft genome length for each of the 27 isolates were compared to the ranges previously reported for *Listeria sensu stricto* and *sensu lato* species. The 25 isolates that clustered with *sensu stricto* have G+C content from 35.9–38.9mol% and draft genome lengths from 2.8 to 3.1 Mb, which is within the ranges (34.6–41.6mol% and 2.8–3.2 Mb) currently described for this clade [18]. Similarly, the two isolates that cluster with *sensu lato* have G+C content of 41.9 and 42.3mol% and draft genome lengths of 3.2 and 3.1 Mb, both within the ranges currently described for this clade (38.3–45.2mol% and 2.6–3.5 Mb) [11–13, 18]. Screening for WGS contamination was completed using Kraken [34]. All draft genomes met the minimum quality standards for taxonomic assessment specified by Chun *et al*. [35]. WGS quality data and NCBI GenBank accession numbers for all 27 isolates can be found in Table S1.

**Fig. 1.**
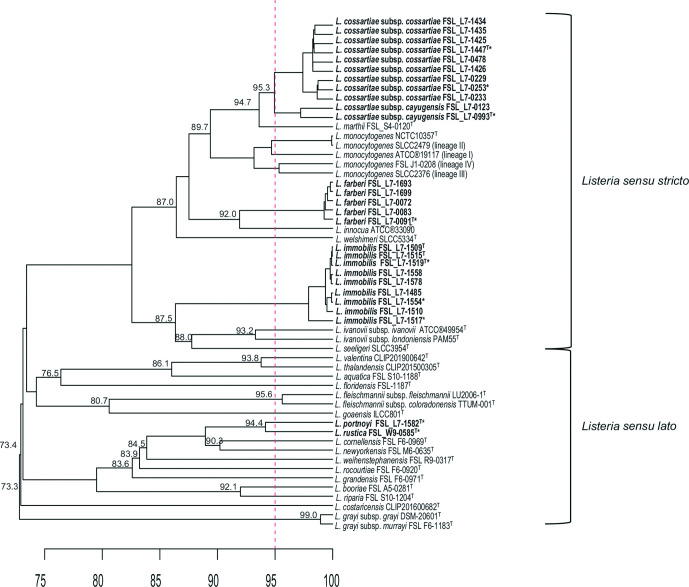
UPGMA hierarchical cluster dendrogram based on the average nucleotide identity blast (ANIb) analysis of all 27 draft genomes representing the five novel *Listeria* species and two subspecies proposed here and 28 reference strains representing 21 *Listeria* species. The vertical bar is placed at 95 corresponding to the proposed 95 % ANIb cut-off for species differentiation [75]. The horizontal scale represents ANI percent similarity. The values placed on nodes represent ANI similarities; for terminal nodes, this value represents the similarity between the two taxa; for internal nodes, the value represents the similarity between the two taxa in the different branches that are most similar to each other. ANI similarities are only shown for nodes that split a given species from one or more other species. The isolates selected for phenotypic characterization are indicated with an ‘*”. The novel species are bolded with type strains identified by a superscript T.

Average nucleotide identity using blast (ANIb) analysis was conducted using the draft genomes for the 27 novel species isolates characterized here and a set of 28 *Listeria* reference genomes (Figs 1 and S1 represents an ANI difference dendrogram containing only type strains). The reference set included one genome for each of the four *L. monocytogenes* lineages along with the type strains for all known species and subspecies characterized as of 29 October 2020. ANIb analysis was completed using pyani version 0.2.7 [36]. The ANI-based dendrogram of the pyani output was created with the dendextend R package [37] (Fig. 1). The ANI analysis showed that the 27 isolates grouped into five distinct clusters, three within the *sensu stricto* clade and two within *sensu lato*; each cluster showed ANI values with <95 % similarity to each other and all known *Listeria* species. *L. farberi* (represented by five isolates) clusters with *L. innocua* (91.9 % ANI between the *L. farberi* type strain and the *L. innocua* reference genome), while *L. immobilis* (represented by nine isolates) clusters closest to *L. ivanovii* subsp. *londoniensis* (ANI of 87.4 % between the *L. immobilis* type strain and the reference genome representing *L. ivanovii* subsp. *londoniensis *; see Fig. 1). *L. cossartiae* (represented by 11 isolates) clusters with *L. marthii* and is proposed to include two subspecies [subsp. *cossartiae* (nine isolates) and subsp. *cayugensis* (two isolates) with an ANI of 93.4 and 94.7 % between the respective type strains and *L. marthii *]; these two proposed subspecies show an average ANI value of 94.9 % similarity to each other with a 95.2 % similarity between the type strains for each subspecies. *L. portnoyi* clusters closest to *L. cornellensis* and *L. newyorkensis* with average ANI values of 88.9 and 89.2 %, respectively. *L. rustica* also clusters closest to *L. cornellensis* and *L. newyorkensis* with average ANI values of 88.9 and 88.7 %, respectively. The maximum ANI value between *L. portnoyi* and *L. rustica* is 94.3 %, which warrants classification as two distinct novel species. The ANI distance matrix for all genomes is provided in supplementary material S7.

**Fig. 2.**
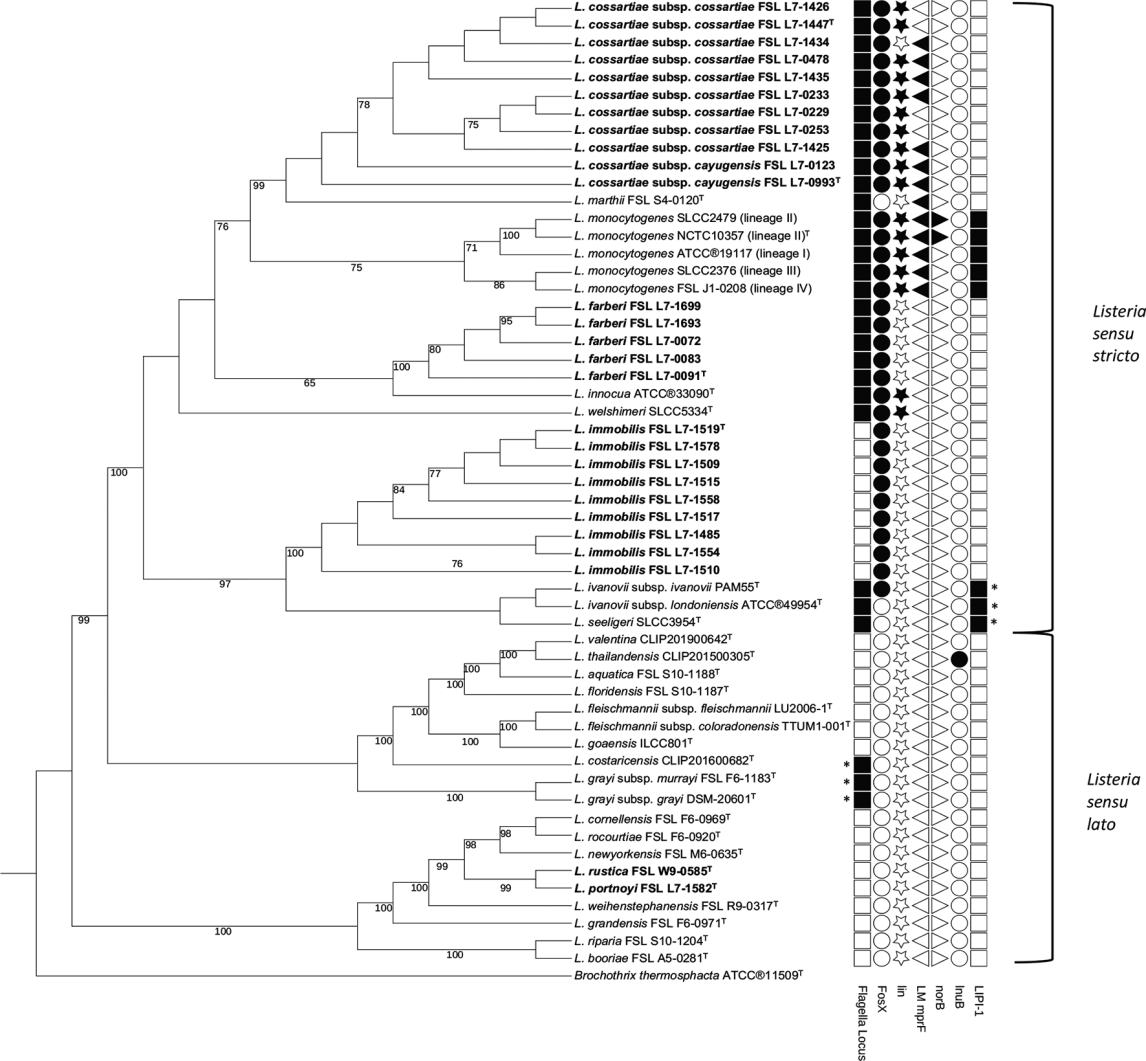
Maximum-likelihood consensus phylogeny based on the concatenation of a 120-bacterial protein marker set (bac120) from GTDB-Tk analysis of the 27 draft genomes representing the five novel *Listeria* species and two subspecies and the same reference set used for ANIb analysis (see Fig. 1). The phylogeny was reconstructed using RAxML v8.2.12 and the PROTGAMMAILGF model. The values on the branches represent bootstrap values based on 1000 replicates; bootstrap values <70 are not shown. The tree is rooted at the midpoint and includes the outgroup *Brochothrix thermosphacta* ATCC®11509^T^. The tree was edited with iTOL v5 [43]. Novel *Listeria* species are bolded and type strains are identified with a superscript T. The presence/absence of key genes, operons, and loci in the draft genomes were mapped onto the tree using iTOL [43] and indicated with filled/unfilled symbols (filled indicates presence, unfilled indicates absence). Symbols with an ‘*’indicate loci containing diversified genes and consequently only some genes were detected using a blastn search with reference *L. monocytogenes* genes from the PasteurMLST databases [61, 62]; specifically (**i**) not all genes in the flagella locus sourced from the cgMLST1748 database (lmo0676 thru lmo0717 [76]; Table S4) were detected in the *L. grayi* and *L. costaricensis* genomes, and (ii) not all LIPI-1 genes sourced from the Virulence database were detected in the *L. ivanovii* and *L. seeligeri* genomes. Detection of diversified flagella and LIPI-1 genes was achieved via alternative search methods including (**i**) locating the genes in the NCBI GenBank annotated genomes, or (ii) using more closely related reference genes (e.g. the *L. ivanovii* prfA gene cluster).

Additional whole-genome based phylogenetic analysis of the type strains for the novel *Listeria* species and subspecies proposed here was performed using the Genome Taxonomy Database Toolkit (GTDB-Tk) as described by Parks *et al*. [38, 39] and Chaumeil *et al*. [40]. GTDB-Tk provides taxonomic assignment of a query bacterial or archaeal genome based on the query genome’s phylogenetic placement in the GTDB-Tk reference tree, its relative evolutionary divergence (RED), and its ANI value to the reference genomes [40]. The 27 novel species draft genomes, as well as the same 28 reference genomes described above, were included in the GTDB-Tk assessment. Unlike the genomes in the reference group, no existing species reference genomes were assigned to the novel species; however, all novel species were placed in the *Listeria* genus, supporting that the strains represent novel *Listeria* species. A phylogenetic tree was inferred using the alignment of 120 bacterial protein marker genes (bac120 [38]) obtained from the GTDB-Tk analysis that included the draft genomes of the novel species strains, the 28 reference genomes, and *Brochothrix thermosphacta* ATCC^®^11509^T^ (incorporated as the outgroup). The best fit model for protein evolution was determined using ProtTest 3.4.2 [41], and the maximum-likelihood tree was inferred using RAxML version 8.2.12 [42] with 1000 bootstrap replicates. A graphical view of the tree was generated using iTOL version 5 [43]; the tree was midpoint-rooted. The GTDB-Tk bac120 phylogenetic placement of the novel species was consistent with that in the ANIb dendrogram (Fig. 2); as with the ANI dendrogram (Fig. 1), three of the novel species are within the *sensu stricto* clade*,* and two are within *sensu lato*. Of the three novel *sensu stricto* species, *L. cossartiae* subsp. *cossartiae* and subsp. *cayugensis* cluster with *L. marthii, L. farberi* clusters with *L. innocua,* and *L. immobilis* represents a sister clade to the clade that includes *L. ivanovii* and *L. seeligeri *. The two novel *sensu lato* species, *L. portnoyi* and *L. rustica,* cluster most closely to *L. newyorkensis* and within a group designated by GTDB as *Listeria *_A, which includes *L. booriae *, *L. cornellensis *, *L. newyorkensis *, *L. grandensis *, *L. riparia *, *L. rocourtiae* and *L. weihenstephanensis *.

**Fig. 3.**
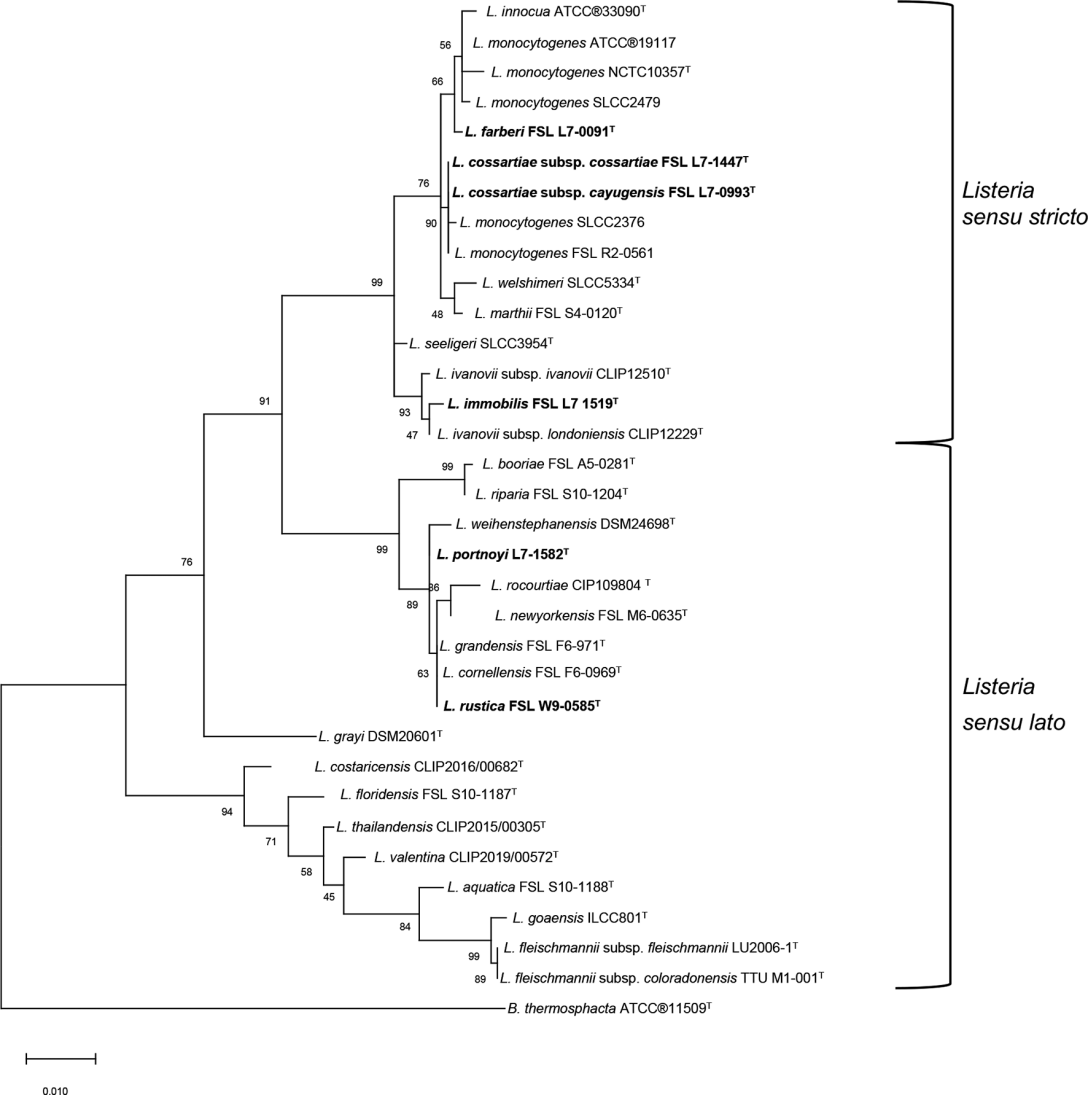
Maximum-likelihood phylogeny based on 16S rRNA sequence analysis of the type strains representing the five novel species and two subspecies using mega X. A total of 1078 positions were included in the dataset. The same reference set used for ANIb analysis was included along with *Brochothrix thermosphacta* ATCC®11509^T^ for an outgroup. The tree was reconstructed using mega X with 1000 bootstrap replicates and the Kimura two-parameter model [50, 51]; bootstrap values <70 are not shown. Novel *Listeria* species type strains are bolded and identified with superscript T.

Additional species classification approaches [i.e. *in silico* DNA–DNA hybridization (*is*DDH), average amino acid identity (AAI) and phylogenetic analysis of the 16S rRNA gene] further support ANI and GTDB-Tk-based identification of the novel species and subspecies described here. The *is*DDH values were determined using the Genome-to-Genome Distance Calculator (GGDC) 2.1, formula 2 [identities/ high-scoring segment pair (HSP)] available from Leibniz Institute DSMZ [44]. The *is*DDH values of the six type strains representing the novel species (and subspecies) described here ranged from 31.8–53.6% when compared to the most similar *Listeria* reference genome (Table 1), well below the DDH cut-off for species delineation (<70 %) [45]. Additionally, the *is*DDH between the *L. cossartiae* subspecies type strains (*L. cossartiae* subsp. *cossartiae* L7-1447^T^ vs. *L. cossartiae* subsp. *cayugensis* L7-0993^T^) is 61.5 %, well below the <79 % criteria proposed for subspecies delineation [46]. Pairwise AAI values were calculated using the online pairwise AAI calculator from the Enve-omics packages with the default parameters [47]. The AAI values between the novel species and the most similar *Listeria* reference genome ranged from 91.0–97.3 % (Table 1). While AAI does not provide good resolution of species that are closely related (ANI similarity between 80–100 %) [47], the AAI values we obtained were all >60 %, which confirms placement within the genus *Listeria* [47]. The 16S rRNA genes of the novel species type strains and the 28 reference genomes described above were aligned using muscle [48]. mega X [49, 50] was used to compute pairwise distances between the 16S rRNA sequences and to infer a maximum-likelihood phylogenetic tree (Fig. 3) using the Kimura two-parameter model [51] and 1000 bootstraps. The highest 16S rRNA gene sequence similarity between each novel species and a reference *Listeria* species is: (i) 99.9 % between *L. cossartiae* (both subspecies *cossartiae and* subsp. *cayugensis*) and *L. monocytogenes* lineage I SLCC2376; (ii) 99.7 % between *L. farberi* and *L. monocytogenes* lineage II SLCC 22479; (iii) 99.9 % between *L. immobilis* and the *L. ivanovii* subsp. *londoniensis* type strain; (iv) 99.9 % between *L. portnoyi* and both the *L. cornellensis* and *L. grandensis* type strains; and (v) 100 % between *L. rustica* and the *L. cornellensis* and *L. grandensis* type strains. While these values are above the proposed species cut-off of 98.7–99.0 % [52], similarities of >99 % between 16S rRNA gene sequences of different species are not uncommon [52]; this high level of similarity is also consistent with previous observations that different *Listeria* species often show highly similar 16S rRNA gene sequences [53, 54]. The 16S rRNA sequence similarity data for the novel species however further supports placement in the genus *Listeria *.

**Table 1.** Whole genome-based comparisons of the novel species to *Listeria* species with the highest similarity

Novel species and subspecies	*is*DDH (%)*	AAI (%)†	ANI (%)‡	Most similar *Listeria* species
*L. cossartiae* subsp. *cossartiae* FSL L7-1447^T^	53.6	96.8	93.4	* L. marthii* FSL S4-120^T^
*L. cossartiae* subsp. *cayugensis* FSL L7-0993^T^	52.6	97.3	94.7	* L. marthii* FSL S4-120^T^
*L. farberi* FSL L7-0091^T^	46.4	95.2	91.9	* L. innocua* ATCC 33090^T^
*L. immobilis* FSL L7-1519^T^	34.3	91.9	87.4	* L. ivanovii* subsp. *londoniensis* ATCC 49954^T^
*L. portnoyi* FSL L7-1582^T^	37.3	91.7	88.9	* L. cornellensis* FSL F6-0969^T^
	38.0	92.2	89.2	* L. newyorkensis* M6-0535^T^
*L. rustica* FSL W9-0585^T^	37.6	91.7	88.7	* L. cornellensis* FSL F6-0969^T^
	37.9	92.6	88.9	* L. newyorkensis* M6-0535^T^
*L. cossartiae* subsp. *cossartiae* FSL L7-1447^T^§	61.5	97.5	95.2	*L. cossartiae* subsp. *cayugensis* FSL L7-0993^T^

*isDDH, *in silico* DNA–DNA hybridization.

†AAI, average amino acid identity from two-way analysis.

‡ANI, average nucleotide identity.

§*L. cossartiae* subspecies comparison performed to support subspecies classification.

## Phenotypic analysis

Phenotypic characterization was performed on nine isolates, which represent the five species and two subspecies. Isolates in this set included: (i) *L. farberi* (*n*=1); (ii) *L. portnoyi* (*n*=1); (iii) *L. rustica* (*n*=1); (iv) *L. immobilis* (*n*=3); (v) *L. cossartiae* subsp. *cossartiae* (*n*=2) and subsp. *cayugensis* (*n*=1). These isolates represented all novel *sensu stricto* type strains, as well as two additional isolates for *L. immobilis* (FSL L7-1517 and FSL L7-1554) and one additional isolate for *L. cossartiae* subsp. *cossartiae* (FSL L7-0253); these additional isolates were selected to ensure better representation of the species and subspecies that included the largest number of isolates, i.e. *L. immobilis* (nine isolates) and *L. cossartiae* subsp. *cossartiae* (nine isolates). Phenotypic analyses conducted on the nine isolates included (i) growth assessment across the growth temperature range expected for *Listeria* (0–45 °C) [55], (ii) assessment of colony phenotypes on selective and differential media, (iii) the *Listeria* identification procedures described in the FDA BAM, Chapter 10 and ISO EN 11290-1 : 2017 (both documents describe the same tests); specific tests conducted here included haemolysis, motility, catalase, oxidase, Gram stain, nitrate reduction, and API *Listeria* (bioMérieux) [26, 56], (iv) growth under anaerobic conditions, and (v) the biochemical tests included in the API 20E (bioMérieux) and API 50CH kits (bioMérieux). Tests that are classically used to differentiate *Listeria* species [26, 56, 57] are included in the API *Listeria* (i.e. rhamnose, xylose) and API 50CH (i.e. mannitol). The phenotypic analyses were conducted in two biological replicates, using pure cultures grown aerobically on BHI agar at 30 °C for 24 h. The positive and negative control strains for phenotypic analyses included the well-characterized *L. monocytogenes* 10403S reference strain [58] or appropriate type strains for other species available from reference culture collections per the requirements of ISO EN 11290-1 : 2017 and ISO 11133 : 2014 [56, 59]; specific control strains used for each test are detailed below.

Growth experiments, with *L. monocytogenes* 10403S as a control, were conducted at 4, 22, 30, 37 and 41 °C. Overnight cultures were grown in 5 ml BHI broth incubated at 30 °C. For each combination of temperature and strain, a 5 ml BHI broth aliquot was inoculated with an overnight culture to yield between 60 and 300 c.f.u. ml^−1^, followed by incubation under static conditions at the temperatures specified above. BHI cultures incubated at 22, 30, 37, and 41 °C were enumerated after incubation for 24 and 48 h; cultures incubated at 4 °C were enumerated after 10 and 14 days. All enumerations were performed by spread-plating in duplicate onto BHI agar followed by incubation at 30 °C for 24–36 h. Changes in bacterial numbers were calculated for each time point relative to the starting concentrations (see Table S2 for detailed results). Bacterial numbers for all seven isolates representing the novel *sensu stricto* species increased by at least 7 logs after either 24 or 48 h of incubation at temperatures between 22 and 41 °C, and 2 logs after 14 days at 4 °C. All *sensu stricto* isolates grew optimally at either 30 or 37 °C after 24 h (indicated by the fact that either of these temperatures yielded the highest log increase in bacterial numbers after 24 h). For the two *sensu lato* isolates, bacterial numbers increased by at least 6 logs after 48 and 24 h of incubation at 22 and 30 °C, respectively, and ≥4 logs after 14 days at 4 °C. *L. rustica* showed an average increase in bacterial numbers of only 1.73 log c.f.u. ml^−1^ after 48 h at 37 °C and no growth at 41 °C. *L. portnoyi* did not grow at either 37 and 41 °C; in all cases of ‘no growth’, bacterial numbers after incubation were lower than the starting inoculum. No growth for a given species/temperature combination was confirmed by (i) additional enumerations every 24 h for up to 5 days for the initial two replicates and (ii) completion of a third biological replicate with enumeration every 24 h over 7 days; the additional experiments consistently showed no growth.

Colony morphology, aesculin hydrolysis and PI-PLC activity was assessed by performing a three-phase streak of overnight BHI broth cultures onto MOX and LMCPM plates. Bacteria positive for aesculin hydrolysis will yield grey to black colonies surrounded by a black halo on MOX. LMCPM is a chromogenic media that detects PI-PLC activity through hydrolysis of the chromogen X-inositol phosphate. *Listeria* species positive for PI-PLC will appear blue-green on LMCPM, and species lacking PI-PLC activity will appear white. All novel species colonies were black, round, surrounded by a black halo and had sunken centres on MOX, and were small, round, convex and white on LMCPM. Blackening on MOX verified that all the novel species hydrolyze aesculin. The absence of blue-green colonies on LMCPM is indicative of a lack of PI-PLC activity. *L. monocytogenes* 10403S and *L. innocua* ATCC^®^33090^T^ were included as positive and negative controls for PI-PLC activity, respectively. Only *L. monocytogenes* generated blue-green colonies. Interestingly, the two isolates representing the novel *sensu lato* species (*L. portnoyi*, *L. rustica*) appeared more sensitive to the selective pressure of LMCPM compared to the seven isolates representing the three novel *sensu stricto* species and the two control strains; growth was limited to approximately ten colonies in the primary streak with the novel *sensu lato* isolates compared to growth in all phases with the novel *sensu stricto* species and the control strains. Overall, the novel species and subspecies reported here could not be differentiated based on their colony appearances.

The novel species were assessed for their ability to reduce nitrate following the BAM/ISO methods using the detailed protocol described by Buxton *et al*. [60]; the ability to reduce nitrite was assessed using the same protocol with nitrite broth used in place of nitrate broth. A heavy inoculum of pure culture growth from BHI agar was added to both nitrite and nitrate broth for each test strain; *L. monocytogenes* 10403S and *L. booriae* FSL A5-0281^T^ were included as negative and positive controls, respectively. Following incubation of nitrite and nitrate broth cultures at 35 °C for 24 h [60], aliquots of each culture were combined with NIT1 and NIT2 (i.e. sulfanilic acid and *N*,*N*-dimethyl-α-napthylamine, bioMérieux); the appearance of a red-violet colour when combined with NIT1 and NIT2 indicates nitrite is present. Powdered zinc (bioMérieux) was added to the nitrate broth cultures that did not change colour to test for the reduction of nitrate to molecular nitrogen (colour change after zinc addition confirms that nitrate has not been reduced). Cultures that were negative for nitrate or nitrite reduction after 24 h were tested again after 5 days of incubation. None of the isolates representing the novel *sensu stricto* species reduced nitrate; the isolates representing the novel *sensu lato* species reduced nitrate as evident by a red-violet colour change after the addition of NIT1 and NIT2. None of the novel species reduced nitrite, as evidenced by the nitrite enrichments developing a red colour when combined with NIT1 and NIT2.

Motility was assessed at two temperatures, 25 and 37 °C. At 25 °C, motility was observed microscopically and via inoculation of motility test medium (MTM; Becton Dickinson). Isolated colonies grown on BHI agar were inoculated into MTM prepared in 10 ml screw-capped tubes, followed by incubation at 25 °C; MTM tubes were checked every 24 h for up to 7 days. Microscopic observations were performed by preparing wet mounts of BHI agar cultures incubated at 25 and 37 °C. *L. monocytogenes* 10403S and *L. booriae* FSL A5-0281^T^ were included as positive and negative controls, respectively. Among the novel *sensu stricto* species, the isolates representing *L. cossartiae* subsp. *cossartiae, L. cossartiae* subsp. *cayugensis* and *L. farberi*, along with the *L. monocytogenes* control, all exhibited the typical motility characteristics at 25 °C (i.e. umbrella-like growth pattern in MTM and microscopic observations of tumbling). The three isolates representing the novel *sensu stricto* species *L. immobilis* (FSL L7-1517, FSL L7-1519^T^, FSL L7-1554) were non-motile at 25 °C. The novel *sensu lato* species and the *L. booriae* control were also non-motile at 25 °C, a characteristic common to all *sensu lato* but *L. grayi* [7] described to date [* L. costaricensis* is reported as motile, but only at 37 °C [11]]. None of the novel species isolates or control strains were motile at 37 °C. Given that all currently described *sensu stricto* species typically exhibit motility at 25 °C [18, 55], further analysis of the draft genomes of all nine isolates representing *L. immobilis* was performed. As described below, the genes associated with motility were not detected in any of the nine *L. immobilis* isolates, confirming the phenotypic motility results.

Gram staining, oxidase activity, catalase activity, β haemolysis, and growth under anaerobic conditions were performed using colonies isolated from fresh BHI agar cultures prepared as described above. All the novel species isolates are oxidase-negative (OxiStrips, Hardy Diagnostics), catalase-positive, Gram-positive, short rods and grow anaerobically. Haemolysis was assessed using sheep blood agar (SBA; Becton Dickinson). *L. monocytogenes* 10403S and *L. booriae* FSL A5-0281^T^ were included as positive and negative controls, respectively. A clear zone around the stab location of the colony was considered positive for β haemolysis. For each biological replicate, two colonies for each novel species isolate were inoculated into SBA. The nine isolates representing the five novel species and the *L. booriae* negative control strain were all non-haemolytic; only L. monocytogenes was haemolytic.

*Listeria* API analyses were conducted according to the manufacturer’s instructions (bioMérieux). Following inoculation using fresh BHI cultures, prepared as described above, the test strips were incubated aerobically at 35 °C. The numeric codes were evaluated using the apiweb database (bioMérieux version 2.0, apiweb version 1.4.0). Overall, the API identifications for novel *sensu stricto Listeria* corresponded to the reference genomes to which these isolates showed the highest ANI similarity. *L. farberi* generated a numeric profile (7510) that is considered a very good species identification; however, it was identified to be *L. innocua *. The numeric profile for *L. immobilis* (3330) was identified as *L. ivanovii* although with a T value <1, due to negative results for glucose-1-phosphate (both *L. ivanovii* subsp. and *L. ivanovii* subsp. *londoniensis* ferment glucose-1-phosphate). Additionally, the lack of haemolysis with *L. immobilis* makes the *L. ivanovii* identification questionable since *L. ivanovii* is hemolytic. *L. cossartiae* subsp. *cossartiae* yielded the same API numerical profile (6110) previously reported for *L. marthii* [6]; as *L. marthii* is not included in the API web database as of 29 October 2020, the actual species assignment obtained by the API web database was *L. monocytogenes* (T value <1). *L. cossartiae* subsp. *cayugensis* API numerical profile (6130) was identified as *L. grayi *, but also with a T value <1. *L. cossartiae* subsp. *cossartiae* and subsp. *cayugensis* generated different numeric profiles due to differences in the ability to ferment ribose (6110 vs. 6130). The differential ribose result between the *L. cossartiae* subspecies was further evaluated by additional testing with ribose fermentation broth as described in the FDA BAM [26]. FSL L7-0123, another isolate representing *L. cossartiae* subsp. *cayugensis* was added to the broth analysis along with FSL L7-0993^T^ and the two isolates (FSL L7-0253, FSL L7-1447^T^) representing *L. cossartiae* subsp. *cossartiae*. The ribose broth fermentation results agreed with the API ribose results for L7-0993^T^ (*L. cossartiae* subsp. cayugensis; utilized ribose) and FSL L7-0253, FSL L7-1447^T^ (*L. cossartiae* subsp. *cossartiae*; did not utilize ribose). However, FSL L7-0123, the other *L. cossartiae* subsp. *cayugensis* isolate did not utilize ribose, which suggests that the ribose phenotype is variable among this subspecies. Analysis of the 11 *L*. *cossartiae* draft genomes for genes involved in ribose utilization supported the phenotypic ribose fermentation results (details described below). Both of the novel *sensu lato* species (*L. portnoyi* and *L. rustica*) generated the same API *Listeria* numeric profile, 2710, which does not provide acceptable identification to the species level; this code was also previously reported for *L. weihenstephanensis* [17]. As with all other currently described *sensu lato* species, excluding *L. grayi *, both *L. portnoyi* and *L. rustica* are negative for *Listeria* API DIM (Differentiation of *L. innocua* and *L. monocytogenes *), which is based on d-arylamidase activity. See Table 2 for a summary of the *Listeria* API numeric codes.

**Table 2.** API *Listeria* numerical profiles and corresponding identification results as reported from the apiweb identification software for the *Listeria* species currently recognized in the FDA BAM and the *Listeria* spp. nov. and subsp. nov.

Strain	API numerical profile*	Significant taxa†	% ID‡	T value§
* Listeria* species currently included in the FDA BAM and *Listeria* API database:				
* L. monocytogenes *	6510	* L. monocytogenes *	98.5	1.0
* L. ivanovii* subsp. *ivanovii *	3370	* L. ivanovii *	99.9	0.92
* L. ivanovii* subsp. *londoniensis *	3350	* L. ivanovii *	99.8	1.0
* L. innocua *	7510	* L. innocua *	99.6	1.0
* L. welshimeri *	7711	* L. welshimeri *	99.9	1.0
* L. seeligeri *	3310	* L. seeligeri *	94.2	1.0
* L. grayi *	7120	* L. grayi *	99.9	1.0
* Listeria* spp. nov. and subsp. nov.:				
*L. farberi* FSL L7-0091^T^	7510	* L. innocua *	99.0	1.0
*L. cossartiae* subsp. *cossartiae* FSL L7-1447^T^	6110	* L. monocytogenes *	80.0	0.62
*L. cossartiae* subsp. *cayugensis* FSL L7-0993^T^	6130	* L. grayi *	99.3	0.5
*L. immobilis* FSL L7-1519^T^	3330	* L. ivanovii *	96.0	0.68
*L. portnoyi* FSL L7-1582^T^	2710	* L. ivanovii *||	59.2	0.21
*L. rustica* FSL W9-0585^T^	2710	* L. ivanovii *	59.2	0.21

*The API *Listeria* identification kit includes 10 tests that assess either enzymatic activity or carbohydrate fermentation. Tests are separated into groups of three and a value of 1, 2 or 4 is assigned to each; the values for positive reactions are added together to create a four-digit numerical profile (the tenth test has a value of 1). The numerical values for the *Listeria* species identified in the FDA BAM represent the most common numerical profile in the API database for a given species (*i.e*. other profiles are possible, but less common). The numerical values for the *Listeria* species and subsp. nov. were determined through duplicate analyses; duplicates always provided the same results.

†‘Significant taxa’ represents the top *Listeria* species match listed on the API report.

‡The % ID is the accuracy of the numerical profile to the species listed under significant taxa based on the historical results from characterizations of strains in the database.

§The T value is an estimate of how closely the numerical profile matches the typical reaction for the species. A T value <1 indicates 1 or more aberrant biochemical reactions for the species in question.

||The numeric profile 2710 result report stated ‘acceptable identification to the genus’.

Isolates were further characterized using API CH50 and API 20E kits, which were conducted per the manufacturer’s instructions (bioMérieux). The protocol for *Bacillus* was followed for the CH50 strip; CHB/E medium was used for inoculation of the API 50 CH strip. For API 20E, the inoculum was prepared in 5 ml of NaCl 0.5 % Medium (bioMérieux). Following inoculation with freshly prepared pure cultures, the API CH50 and API 20E test strips were incubated aerobically at 30 and 35 °C, respectively. The Voges–Proskauer reaction was assessed 10 min after the addition of VP1 and VP2 reagents (bioMérieux) to the VP test well on the API 20E strip. Reactions included in the API CH50 allowed for phenotypic differentiation of (i) *L. cossartiae* subsp. *cayugensis* from *L. marthii *, *(ii) L. farberi* from *L. innocua *, and (iii) *L. portnoyi* and *L. rustica* from each other as well as from *L. weihenstephanensis *. Details regarding the differentiating characteristics are provided in the species descriptions and a summary of the phenotypic results can be found in Tables 3 and S3.

**Table 3.** Summary of the phenotypic characteristics of the novel *Listeria* species compared to previously reported characteristics of other species Taxa: Lcs, *L. cossartiae* (this study); Lfr, *L. farberi* (this study); Lim, *L. immobilis* (this study); Lpo, *L. portnoyi* (this study); Lru, *L. rustica* (this study); Lmo, *L. monocytogenes* [15, 23]; Lma, *L. marthii* [6, 23]; Lin, *L. innocua* [15, 23]; Lws, *L. welshimeri* [15, 23]; Liv, *L. ivanovii* [4, 15, 23]; Lse, *L. seeligeri* [15, 23]; Lgy, *L. grayi* [15, 23]; Lfc, *L.fleischmannii* [7, 8, 10]; Lgo, *L. goaensis* [12]; Lfl, *L. floridensis* [10]; Lth, *L. thailandensis* [13]; Lva, *L. valentina* [14]; Lco, *L. costaricensis* [11]; Laq, *L. aquatica* [10]; Lny, *L. newyorkensis* [15]; Lcn, *L. cornellensis* [10]; Lro, *L. rocourtiae* [1, 8]; Lwp, *L. weihenstephanensis* [10, 17]; Lgd, *L. grandensis* [10]; Lri, *L. riparia* [10]; Lbo, *L. booriae* [15]. +, Positive; (+), weak positive; V, variable between replicates and/or strains; V!, variable between studies; V†, *L. cossartiae* subsp. *cossartiae* does not ferment ribose and subsp. *cayugensis* strains are variable for ribose fermentation; V*, characteristic that differentiates subspecies; *L. ivanovii* subsp. *ivanovii* ferments ribose while subsp. *londoniensis* does not ferment ribose; *L. grayi* subsp. *grayi* does not reduce nitrate and ferments methyl α-d-glucopyranoside, while subsp. *murrayi* reduces nitrate and does not ferment methyl α-d-glucopyranoside; *L. fleischmannii* subsp. *fleischmannii* ferments turanose, while subsp. *coloradonensis* does not ferment turanose; (α), alpha haemolysis observed; PI-PLC, phosphoinositide phospholipase C.

	Novel species					Species described as of 5 October 2020																				
Characteristic	*sensu stricto*			*sensu lato*		*sensu stricto*						*sensu lato*														
	Lcs	Lfr	Lim	Lpo	Lru	Lmo	Lma	Lin	Lws	Liv	Lse	Lgy	Lfc	Lgo	Lfl	Lth	Lva	Lco	Laq	Lny	Lcn	Lro	Lwp	Lgd	Lri	Lbo
Voges–Proskauer	+	+	+	−	−	+	+	+	+	+	+	+	−	−	−	+	−	+	V	−	−	−	−	−	−	−
Nitrate reduction	−	−	−	+	+	−	−	−	−	−	−	V*	+	−	−	+	−	+	+	+	+	+	+	+	+	+
Motility	+	+	−	−	−	+	+	+	+	+	+	+	−	−	−	−	−	+	−	−	−	−	−	−	−	−
Haemolysis	−	−	−	−	−	+	−	−	−	+	+	−	−	+ (α)	−	−	−	−	−	−	−	−	−	−	−	−
PI-PLC	−	−	−	−	−	+	−	−	−	+	−	−	−	−	−	−	−	−	−	−	−	−	−	−	−	−
d-Arylamidase	−	+	+	−	−	−	−	+	V	V	+	+	−	−	−	−	−	−	−	−	−	−	−	−	−	−
⍺-Mannosidase	+	+	−	−	−	+	+	+	+	−	−	V	−	−	−	−	−	−	+	−	−	+	−	−	+	+
d-Arabitol	+	+	+	(+)	(+)	+	+	+	+	+	+	+	+	+	−	+	+	+	−	−	−	−	+	V	−	+
d-Xylose	−	−	+	+	+	−	−	−	+	+	+	−	+	+	+	+	+	+	+	+	+	+	+	+	+	+
l-Rhamnose	−	+	−	+	+	+	−	V	V	−	−	−	+	+	+	+	+	+	+	V	−	+	+	−	+	+
Methyl ⍺-d-Glucopyranoside	+	+	+	+	+	+	+	+	+	+	+	V*	+	+	+	+	−	+	−	+	+	+	+	+	+	+
Methyl ⍺-d-Mannopyranoside	+	+	−	−	−	+	+	+	+	−	−	+	−	−	−	−	−	+	+	−	−	+	−	−	+	+
d-Ribose	V†	−	+	−	−	−	−	−	−	V*	−	+	+	−	−	+	+	+	+	+	+	+	−	+	V	V
Glucose-1-Phosphate	−	−	−	−	−	−	−	−	−	V	−	−	−	−	−	−	−	−	−	−	−	−	−	−	−	−
d-Tagatose	−	−	−	−	−	−	−	−	+	−	−	−	−	−	−	−	V	+	+	−	−	−	−	−	−	−
Glycerol	+	−	V	−	−	V	−	+	+	+	+	V	+	(+)	−	(+)	+	+	V	+	V	+	+	−	V	^+^
l-Arabinose	−	−	−	−	+	−	−	−	−	−	−	−	−	−	+	−	+	−	+	+	V	−	−	−	+	+
d-Galactose	−	−	−	+	+	V	−	−	−	V	−	+	+	−	^+^	−	−	+	−	+	−	+	−	−	+	+
d-Glucose	+	+	+	+	+	V!	V!	V!	+	V!	+	+	+	+	+	+	+	+	+	+	+	+	+	+	+	+
l-Sorbose	−	−	−	−	−	V!	V!	V!	−	V!	−	V!	V!	−	−	−	−	−	−	−	−	−	−	−	−	−
Inositol	−	−	−	−	−	−	−	−	−	−	−	−	V	−	−	+	+	−	V	−	−	−	−	−	V	−
d-Mannitol	−	−	−	+	+	−	−	−	−	−	−	+	V	−	−	−	−	−	−	+	−	+	+	−	V	+
Maltose	+	+	V	−	+	+	+	+	+	+	+	+	+	+	+	−	−	+	−	+	+	+	+	+	+	+
Lactose	+	+	V	(+)	+	+	+	+	+	+	+	+	+	+	+	−	−	+	−	+	(+)	+	V!	−	+	+
Melibiose	−	−	−	−	−	V!	V!	V	−	−	−	−	V	−	−	−	−	−	−	−	−	+	−	−	V	+
Sucrose	−	−	V	−	−	+	−	+	+	+	+	−	V	−	−	−	−	+	−	−	−	−	−	−	−	−
Inulin	−	−	−	−	−	V!	−	V!	−	−	−	−	−	−	−	−	−	−	−	−	−	−	−	−	−	−
Melezitose	−	−	V	−	−	V	−	V	V	V	V	−	V	−	−	−	−	−	−	−	−	−	−	−	−	−
Turanose	−	−	−	−	−	−	+	V	−	−	−	−	V*	−	−	−	−	−	−	−	−	−	−	−	−	−
d-Lyxose	−	−	−	−	−	V	−	V	V	−	−	V	−	−	+	−	+	−	V	−	−	−	−	−	−	−

## Additional genomic characterization

The 27 draft genomes representing the novel species reported here were queried for the presence of flagellar and virulence genes, using blastn, against reference databases of flagellar and virulence genes. The reference sequences for (i) flagellar genes (see Table S4) and (ii) virulence genes were downloaded from the cgMLST1748 and Virulence schemes, respectively, in the Institut Pasteur open access BIGSDB-*Lm* databases described by Moura *et al*. and Ragon *et al*. [61, 62]. Neither virulence genes (*prfA*, *plcA*, *hly*, *mpl*, *actA* and *plcB*) in the *Listeria* pathogenicity island 1 (LIPI-1) nor the internalin genes *inlA* or *inlB* were detected in any of the 27 draft genomes, indicating the five novel species are non-pathogenic, and indicating no need for virulence testing via an animal model (e.g. mouse virulence assay). Furthermore, none of the 26 *Listeria* flagellar motility genes were detected in the nine draft genomes representing *L. immobilis*, which was phenotypically non-motile. Consistent with the fact that representative isolates of the other two novel *sensu stricto* species (*L. cossartiae* and *L. farberi*) were phenotypically motile, the draft genomes for all isolates representing these species contain the full complement of the flagellar genes typical for motile *Listeria sensu stricto* species. None of the flagellar motility genes were detected in the non-motile novel *sensu lato* draft genomes.

We also performed an *in silico* assessment to determine how the novel species isolates described here would be characterized by the multiplex PCR *L. monocytogenes* serovar analysis procedure described by Doumith *et al*. [63]. Briefly, a blastn query was performed for each of the five sequences targeted by the PCR assay (*lmo0737*, *lmo118*, ORF2819, ORF2110, *prs*) against the 27 novel species type draft genomes; the reference sequences for these genes were obtained from the same Institut Pasteur database described above. All novel species draft genomes contain *prs*, which is expected in all *Listeria* species [63]. Interestingly, the draft genomes for four of the five *L. farberi* isolates (FSL L7-0072, FSL L7-0091^T^, FSL L7-1693, FSL L7-1699) and eight of the nine *L. immobilis* isolates (L7-1485, L7-1509, L7-1510, L7-1515, L7-1519^T^, L7-1554, L7-1558, L7-1578) also contain ORF2110. Analysis of the ORF2110 sequences using the NCBI Primer-blast tool [64] and the primers described by Doumith *et al*. [63] suggests that the *L. immobilis* ORF2110 sequences would not be amplified by these primers (three mismatches for the forward and two mismatches for the reverse ORF2110 primers). Conversely, all of the *L. farberi* sequences had no mismatches against the ORF2110 primers suggesting these isolates would be amplified; detection of this gene would classify isolates as *L. monocytogenes* serovars 4b, 4d, and 4e. These findings suggest the four isolates representing *L. farberi* could be misclassified as *L. monocytogenes *.

The draft genomes of the novel Listeria species were screened for putative, functional antimicrobial resistance genes using the Comprehensive Antimicrobial Resistance Database (CARD 3.1.0) and the Resistance Gene Identifier (RGI 5.1.1) with the criteria for perfect and strict hits only [65]. The draft genomes for the three *sensu stricto* novel species described here all yielded a strict hit for at least one putative AMR protein-coding gene, whereas the *sensu lato* draft genomes yielded no hits. More specifically, RGI yielded hits for: (i) *FosX* with all *L. farberi* and *L. immobilis* draft genomes; (ii) *FosX*, *lin* and *L monocytogenes mprF* with *L. cossartiae* subsp. *cayugensis* and four of the *L. cossartiae* subsp. *cossartiae* draft genomes; and (iii) *FosX* and *lin* or *FosX* and *L. monocytogenes mprF* with the five other *L. cossartiae* subsp. *cossartiae* draft genomes (see Table S5 for AMR gene details). Using the Institut Pasteur detergent resistance gene database (*qac*, *bcrABC*, *ermE*) [61], we queried the draft genomes using blastn; no detergent resistance genes (which have been reported as conferring reduced quaternary ammonium sensitivity [66]) were detected. The draft genomes were also analysed for prophage sequences using phaster [67], which assigns completeness scores based on the proportion of phage genes present (i.e. intact >90, questionable 70–90 and incomplete <70). All of the novel species except *L. rustica* contained at least a questionable phage sequence, and intact sequences were detected in at least one draft genome representing *L. cossartiae* subsp. *cayugensis*, *L. farberi*, *L. immobilis* and *L. portnoyi*. The presence of plasmid sequences in the draft genomes was analysed using Platon [68]. Putative plasmid sequences were identified in at least one draft genome representing each of the three novel *sensu stricto* species proposed here. Plasmid sequences were not detected in any of the proposed novel *sensu lato* species. Phage and plasmid screening results are available in Table S5. We searched the 11 draft genomes representing *L. cossartiae* for d-aminopeptidase coding sequences, which convey d-arylamidase activity [69], to support the phenotypically observed absence of d-arylamidase activity (i.e. DIM-negative with *Listeria* API); the search included a reference genome, *L. innocua* ATCC^®^33090^T^, known to possess d-arylamidase activity. None of the draft genomes representing *L. cossartiae* subsp. *cossartiae* (*n*=9) or subsp. *cayugensis* (*n*=2) contained any homologues for this d-aminopeptidase coding sequences, while the *L. innocua* d-arylamidase positive reference genome yielded a match with a high degree of identity (99.3%); this supports the d-arylamidase-negative phenotype for *L. cossartiae*. To confirm the phenotypic ribose fermentation results, we initially analysed the genome for the ribose positive *L. cossartiae* subsp. *cayugensis* strain FSL L7-0993^T^ and found that it encodes proteins in the phosphoketolase pathway (which allows bacteria to ferment ribose), including ribokinase (protein ID MBC1806461.1), ribose-5-phosphate isomerase A (protein ID MBC1806046.1), ribose-5-phosphate isomerase B (protein ID MBC1806331.1 and MBC1806322.1), ribulose-phosphate 3-epimerase (protein ID MBC1806321.1, MBC1806319.1 and MBC1807987.1) and transketolase (protein ID MBC1805730.1 and MBC1806320.1). *L. cossartiae* subsp. *cayugensis* strain FSL L7-0123, which was subsequently confirmed to not ferment ribose, as well as all nine draft genomes representing *L. cossartiae* subsp. *cossartiae*, including FSL L7-0253 and FSL L7-1447^T^, which were phenotypically shown to not utilize ribose, lack a ribokinase homolog (which represents the first step of the phosphoketolase pathway). These genomics results confirm our phenotypic data.

## Practical significance and discussion

This paper reports five new *Listeria* species, including three new species classified into a *Listeria* clade designated by some as ‘*sensu stricto*’ [18, 70], reflecting that this group consists of the *Listeria* species most closely related to *L. monocytogenes *, a known foodborne pathogen. Detection of non-*monocytogenes sensu stricto* species is considered an indicator of an increased risk for *L. monocytogenes* contamination [71, 72]. By expanding the *sensu stricto* clade, this discovery increases the set of species monitored in food processing and production environments as a measure to prevent contamination and foodborne outbreaks [73]. The findings from this study identified several *Listeria* species attributes of practical significance including (i) the discovery of a non-motile *sensu stricto* (*L. immobilis*), leading to motility no longer being a hallmark phenotype for this clade; (ii) phenotypic characteristics of *L. farberi* and *L. cossartiae* that make these species difficult to differentiate from *L. innocua* and *L. marthii *, respectively; (iii) the presence of ORF2110 sequence in the *L. farberi* genome, which could result in misclassification of this species as *L. monocytogenes* serovar 4b with a previously reported molecular serotyping assay [63]; and (v) reduced or no growth of the novel *sensu lato* species described here at incubation temperatures above 30 °C, which may reduce or prevent recovery of these species with currently used standard methods that include incubation at 35 and 37 °C. Important implications of our findings and the expanded *Listeria* diversity reported here include a need to revise and update phenotypic methods used to identify and speciate *Listeria* isolates. Specifically, the classic tests currently employed by reference methods (e.g. FDA BAM, Health Canada and ISO [26, 56, 57]), to identify *Listeria* species need to include more discriminating carbohydrate utilization tests and specify that not all *sensu stricto* species are motile to prevent possible misclassification. Additionally, rapid methods are often used for *Listeria* species detection, the inclusion of representatives for these novel *sensu stricto* species in inclusivity panels for method validation will be important [74].

## Description of *Listeria cossartiae* sp. nov.

*Listeria cossartiae* (cos. sar'ti.ae. N.L. gen. fem. n. *cossartiae* named in honour of Dr. Pascale Cossart for her research contributions toward our understanding of *Listeria monocytogenes* virulence).

*L. cossartiae* exhibits growth characteristics typical of non-pathogenic *sensu stricto Listeria* species. Gram-positive short rods. Oxidase-negative. Catalase-positive. Facultative anaerobe. Presumed to be non-pathogenic based on the absence of haemolysis on SBA, lack of PI-PLC activity on LMCPM and the absence of six virulence genes (*prfA*, *plcA*, *hly*, *mpl*, *actA* and *plcB*) located on LIPI-1 as well as the absence *inlA* and *inlB*. Colonies on MOX are small, round, black, with sunken centres. Colonies on LMCPM are of similar size as colonies on MOX, round and opaque-white in colour. Displays umbrella-patterned motility in MTM incubated at 25 °C. Tumbling motility observed microscopically at 25 °C. Non-motile at 37 °C. Growth occurs from 4–41 °C in BHI broth with optimal growth between 30–37 °C. Does not reduce nitrate or nitrite. Voges–Proskauer positive. Negative for d-arylamidase and positive for α-mannosidase activity. Does not ferment d-xylose, l-rhamnose, glucose-1-phosphate, d-tagatose, l-arabinose, d-galactose, l-sorbose, inositol, d-mannitol, melibiose, sucrose, inulin, melezitose, turanose or d-lyxose. Positive for fermentation of d-arabitol, methyl α-d-glucopyranoside, methyl α-d-mannopyranoside, glycerol, d-glucose, maltose and lactose. Ribose fermentation is the differentiating phenotypic characteristic between the type strains for the proposed subspecies. Phenotypic differentiation from *L. marthii* is achieved by the ability of both *L. cossartiae* subspecies to utilize glycerol and the inability to ferment turanose. See Table 3 for additional details on the biochemical characteristics differentiating *L. cossartiae* from other *Listeria* species. See Table S3 for additional biochemical results.

## Description of *Listeria cossartiae* subsp. *cossartiae* subsp. nov.

*Listeria cossartiae* subsp. *cossartiae* shows the phenotypic characteristics described above for *L. cossartiae*; while nine strains of this subspecies characterized here are unable to ferment ribose, subsp. *cayugensis* is ribose-variable.

The type strain, FSL L7-1447^T^ (CCUG 74667^T^=LMG 31919^T^) was isolated from soil collected in Alabama, USA on 13 October 2018. The total length of the draft genome of the type strain is 2.8 Mb with a G+C content of 38.7 mol%.

## Description of *Listeria cossartiae* subsp. *cayugensis* subsp. nov.

*Listeria cossartiae* subsp. *cayugensis* (ca. yug. en’sis. N.L. fem. adj. *cayugensis* of or belonging to Cayuga, specifically a reference to Cayuga Lake, one of the Finger Lakes in Central New York, and adjacent to Ithaca, USA, where Cornell University is located).

Growth and non-pathogenic characteristics are identical to *L. cossartiae* subsp. *cossartiae* described above except for the fact that some strains in this subspecies (i.e. the type strain FSL-L7-0993^T^) have the ability to ferment ribose. Importantly, genomic data strongly supports the subspecies distinction, including (i) a maximum ANI between two isolates from the different subspecies of 95.3 % (close to the 95 % species cut-off) and (ii) an average *is*DDH between the subspecies of 61.3 % and a maximum *is*DDH between two isolates in different subspecies of 61.9 %, both well below the proposed 79 % cut-off for subspecies. See Table S6 for *is*DDH pairwise results between each isolate representing the two subspecies.

The type strain, FSL L7-0993^T^ (CCUG 74670^T^=LMG 31918^T^) was isolated from soil collected in Georgia, USA on 8 August 2018. The total length of the draft genome assembly of the type strain is 2.8 Mb with a G+C content of 38.6 mol%.

## Description of *Listeria farberi* sp. nov.

*Listeria farberi* (far'be.ri. N.L. gen. masc. n. *farberi* named in honour of Dr. Jeff Farber for his contributions to both our understanding of *Listeria* and the advancement of food safety).

*L. farberi* exhibits growth characteristics typical of non-pathogenic *sensu stricto Listeria* species Gram-positive short rods. Oxidase-negative. Catalase-positive. Facultative anaerobe. Presumed to be non-pathogenic based on the absence of haemolysis on SBA, lack of PI-PLC activity on LMCPM, and the absence of six virulence genes (*prfA*, *plcA*, *hly*, *mpl*, *actA* and *plcB*) located on LIPI-1 as well as the absence *inlA* and *inlB*. Colonies on MOX are small, round, black, with sunken centres. Colonies on LMCPM were of similar size and shape as colonies on MOX and are opaque-white in colour. Classic umbrella-patterned motility in MTM incubated at 25 °C. Tumbling motility observed microscopically at 25 °C. Non-motile at 37 °C. Growth occurs at 4–41 °C in BHI broth with optimal growth between 30–37 °C after 24 h. Does not reduce nitrate or nitrite. Voges–Proskauer-positive. Positive for d-arylamidase and α-mannosidase activity. Does not ferment d-xylose, d-ribose, glucose-1-phosphate, glycerol, d-tagatose, l-arabinose, d-galactose, l-sorbose, inositol, d-mannitol, melibiose, sucrose, inulin, melezitose, turanose or d-lyxose. Positive for fermentation of d-arabitol, l-rhamnose, methyl α-d-glucopyranoside, methyl α-d-mannopyranoside, d-glucose, maltose and lactose. Differentiation from *L. innocua* is achieved by the lack of glycerol utilization and inability to ferment sucrose. See Table 3 for additional details on the biochemical characteristics differentiating *L. farberi* from other *Listeria* species. See Table S3 for additional biochemical results.

The total length of the draft genome assembly is 3.0 Mb with a G+C content of 36.8 mol%. The type strain, FSL L7-0091^T^ (LMG 31917^T^=CCUG 74668^T^) was isolated from soil collected in Texas, USA on 6 May 2018.

## Description of *Listeria immobilis* sp. nov.

*Listeria immobilis* (im. mo’bi.lis. N.L. fem. adj. *immobilis* ‘non-motile’ named for the species distinct lack of motility, an atypical characteristic of *sensu stricto Listeria* species).

*L. immobilis* exhibits growth characteristics typical of non-pathogenic *sensu stricto Listeria* species except for motility. Non-motile at 25 and 37 °C. Flagella genes absent in the draft genome. Gram-positive short rods. Oxidase-negative. Catalase-positive. Facultative anaerobe. Presumed to be non-pathogenic based on the absence of haemolysis on SBA, lack of PI-PLC activity on LMCPM, and the absence of six virulence genes (*prfA*, *plcA*, *hly*, *mpl*, *actA* and *plcB*) located on LIPI-1 as well as the absence *inlA* and *inlB*. Colonies on MOX are small, round, black, with sunken centres. Colonies on LMCPM were of similar size and shape as colonies on MOX and are opaque-white in colour. Growth occurs at 4–41 °C in BHI broth with optimal growth between 30–37 °C after 24 h. Does not reduce nitrate or nitrite. Voges–Proskauer-positive. *L. immobilis* is positive for d-arylamidase and negative for α-mannosidase activity. Does not ferment l-rhamnose, glucose-1-phosphate, methyl α-d-mannopyranoside, tagatose, l-arabinose, d-galactose, inositol, d-mannitol, melibiose, inulin, turanose or d-lyxose. Able to ferment d-arabitol, d-xylose, methyl α-d-glucopyranoside, d-ribose and d-glucose. *L. immobilis* is variable for the fermentation of glycerol, maltose, lactose, sucrose and melezitose. *L. immobilis* is differentiated from other *sensu stricto* species by the lack of motility. Differentiated from *L. ivanovii* by lack of haemolysis and lack of PI-PLC activity. See Table 3 for details on the biochemical characteristics differentiating *L. immobilis* from other *Listeria* species. See Table S3 for additional biochemical results.

The total length of the draft genome assembly of the type strain is 3.1 Mb with a G+C content of 35.9 mol%. The type strain, FSL L7-1519^T^ (CCUG 74666^T^=LMG 31920^T^), was isolated from soil collected in Wyoming, USA on 10 October 2018.

## Description of *Listeria portnoyi* sp. nov.

*Listeria portnoyi* (port. noy'i. N.L. gen. masc. n. *portnoyi* named in honour of Dr. Daniel Portnoy for his contributions to our understanding of *L. monocytogenes* virulence and pathogenicity).

Gram-positive short rods. Oxidase-negative. Catalase-positive. Facultative anaerobe. Presumed to be non-pathogenic due to the absence of haemolysis, lack of PI-PLC activity and the absence of six virulence genes (*prfA*, *plcA*, *hly*, *mpl*, *actA* and *plcB*) located on LIPI-1 as well as the absence of *inlA* and *inlB*. Colonies on MOX are round, black, and have sunken centres following incubation at 35 °C for 48 h. Colonies on LMCPM are small, round, convex and white following incubation at 35 °C for 48 h. Growth occurs between 4 and 37 °C in BHI broth with optimal growth at 30 °C after 24 h. Non-motile at 25 and 37 °C. Differentiated Voges–Proskauer-negative. Able to reduce nitrate. Does not reduce nitrite. *L. portnoyi* is negative for d-arylamidase and α-mannosidase activity. Does not ferment d-ribose, glucose-1-phosphate, methyl α-d-mannopyranoside, d-tagatose, glycerol, l-arabinose, l-sorbose, inositol, maltose, melibiose, sucrose, inulin, melezitose, turanose or d-lyxose. Able to ferment d-arabitol, d-xylose, l-rhamnose, methyl α-d-glucopyranoside, d-galactose, d-glucose, d-mannitol and lactose. Differentiated from *L. rustica* by the inability to ferment l-arabinose and maltose. *L. portnoyi* also exhibited a narrower range of growth temperatures compared to *L. rustica* with growth occurring between 4 and 30 °C, and optimal recovery at 30 °C after 24 h from BHI broth. Differentiated from *L. weihenstephanensis* by the inability to utilize glycerol and maltose and the ability to ferment d-galactose. See Table 3 for additional details on the biochemical characteristics differentiating *L. portnoyi* from other *Listeria* species. See Table 3 for details on the biochemical characteristics differentiating *L. portnoyi* from other *Listeria* species. See Table S3 for additional biochemical results.

The total length of the draft genome for the type strain is 3.2 Mb with a G+C content of 41.9 mol%. The type strain, FSL L7-1582^T^ (=CCUG 74671^T^=LMG 31921^T^) was isolated from soil collected in South Dakota, USA on 14 October 2018.

## Description of *Listeria rustica* sp. nov.

*Listeria rustica* (rus'ti.ca. L. fem. adj. *rustica* ‘of rural origin’ named to commemorate the rural location from which this species was isolated).

Gram-positive short rods. Oxidase-negative. Catalase-positive. Facultative anaerobe. Presumed to be non-pathogenic due to the absence of haemolysis, lack of PI-PLC activity and the absence of six virulence genes (*prfA*, *plcA*, *hly*, *mpl*, *actA* and *plcB*) located on LIPI-1 as well as the absence of *inlA* and *inlB*. Colonies on MOX are round, black, and have sunken centres following incubation at 35 °C for 48 h. Colonies on LMCPM are small, round, convex and white following incubation at 35 °C for 48 h. Growth occurs between 4 and 37 °C in BHI broth with optimal growth at 30 °C after 24 h. Non-motile at 25 and 37 °C. Voges–Proskauer-negative. Able to reduce nitrate. Does not reduce nitrite. *L. rustica* is negative for d-arylamidase and α-mannosidase activity. Does not ferment d-ribose, glucose-1-phosphate, methyl α-d-mannopyranoside, d-tagatose, glycerol, l-sorbose, inositol, melibiose, sucrose, inulin, melezitose, turanose or d-lyxose. Able to ferment d-arabitol, d-xylose, l-rhamnose, methyl α-d-glucopyranoside, l-arabinose, d-galactose, d-glucose, d-mannitol, maltose and lactose. Differentiated from *L. portnoyi* by the ability to ferment l-arabinose and maltose. Differentiated from *L. weihenstephanensis* by the inability to utilize glycerol and the ability to ferment l-arabinose and d-galactose. See Table 3 for additional details on the biochemical characteristics differentiating *L. portnoyi* from other *Listeria* species. See Table S3 for additional biochemical results.

The total length of the draft genome of the type strain is 3.1 Mb with a G+C content of 42.3 mol%. The type strain, FSL W9-0585^T^ (=CCUG 74665^T^=LMG 31922^T^) was isolated from a stream used to source irrigation water in the Finger Lakes region of New York, USA; the sample was collected on 11 August 2017.

## Supplementary Data

Supplementary material 1

Supplementary material 2
