# Corrigendum: *Listeria cossartiae* sp. nov., *Listeria farberi* sp. nov., *Listeria immobilis* sp. nov., *Listeria portnoyi* sp. nov. and *Listeria rustica* sp. nov., isolated from agricultural water and natural environments

**DOI:** 10.1099/ijsem.0.004885

**Published:** 2021-06-28

**Authors:** Catharine R. Carlin, Jingqiu Liao, Daniel L. Weller, Xiaodong Guo, Renato Orsi, Martin Wiedmann

**Affiliations:** ^1^​ Department of Food Science, Cornell University, Ithaca, NY 14853, USA; ^2^​ Department of Microbiology, Cornell University, Ithaca, NY 14853, USA; ^†^​ Present address: Department of Systems Biology, Columbia University, NY 10032, New York, USA; ^‡^​ Present address: Department of Environmental and Forest Biology, SUNY College of Environmental Science and Forestry, NY 13210, Syracuse, USA

In the published version of this article, the title was listed incorrectly. The title should have read as follows:

*‘Listeria cossartiae* sp. nov., *Listeria farberi* sp. nov., *Listeria immobilis* sp. nov., *Listeria portnoyi* sp. nov., and *Listeria rustica* sp. nov. isolated from agricultural water and natural environments.’

Additionally, the author’s name ‘Dan Weller’ should have been listed as ‘Daniel L. Weller’. The author line should have read as follows:

‘Catharine R. Carlin^1^, Jingqiu Liao^1,2^, Daniel L. Weller^1^, Xiaodong Guo^1^, Renato Orsi^1^ and Martin Wiedmann^1^’

The authors apologise for any inconvenience caused.

