# Supplementary material for: Listeria cossartiae sp. nov., Listeria immobilis sp. nov., Listeria portnoyi sp. nov. and Listeria rustica sp. nov., isolated from agricultural water and natural environments
Source: Int J Syst Evol Microbiol. 2021 May 17;71(5):004795. doi: 10.1099/ijsem.0.004795 (PMC8289207; doi:10.1099/ijsem.0.004795)
Supplement: Supplementary material 1 [file ijsem-71-4795-s001.pdf]

Table S1. Geographic location, sequence quality, and NCBI accession data for the 27 novel *Listeria* species isolates

Novel Species	Isolate	Geographic Location			Sequence Quality Data					NCBI Accession Numbers		
		State	Latitude	Longitude	Total Length (Mb)	G+C content	No. of Contigs	N50 length	Average Coverage	SRA*	Genome Assembly†	16S rRNA#
L. cossartiae subsp. *cayugensis*	FSL L7-0123	NC	35.9370325	-79.0672078	2.83	38.56	9	523254	170	SRR11343877	JAARZC000000000	
L. cossartiae subsp. *cayugensis*	FSL L7-0993^{T}	GA	34.657462	-84.106025	2.82	38.61	29	335203	96	SRR11343939	JAASUV000000000	MT117897
L. cossartiae subsp. *cossartiae*	FSL L7-0229	AL	33.3367219	-86.7407791	2.82	38.67	13	523834	135	SRR11344217	JAARYM000000000	
L. cossartiae subsp. *cossartiae*	FSL L7-0233	AL	33.3367219	-86.7407791	2.87	38.63	11	1439256	176	SRR11344213	JAARYJ000000000	
L. cossartiae subsp. *cossartiae*	FSL L7-0253	AL	33.3435544	-86.7345114	2.80	38.75	12	522584	148	SRR11344210	JAARYG000000000	
L. cossartiae subsp. *cossartiae*	FSL L7-0478	AL	33.4517	-85.85	2.75	38.88	10	1409620	41	SRR11344101	JAASWP000000000	
L. cossartiae subsp. *cossartiae*	FSL-L7-1425	AL	33.1864	-86.1561	2.78	38.8	14	495198	102	SRR11343896	JAASUM000000000	
L. cossartiae subsp. *cossartiae*	FSL-L7-1426	AL	33.1864	-86.1561	2.87	38.64	30	494483	89	SRR11343895	JAASUL000000000	
L. cossartiae subsp. *cossartiae*	FSL-L7-1434	AL	33.1909	-86.1508	2.80	38.8	15	505744	100	SRR11343892	JAASUK000000000	
L. cossartiae subsp. *cossartiae*	FSL-L7-1435	AL	33.1909	-86.1508	2.85	38.68	126	337809	100	SRR11343891	JAASUJ000000000	
L. cossartiae subsp. *cossartiae*	FSL-L7-1447^{T}	AL	33.1962	-86.1388	2.82	38.73	16	1417199	86	SRR11343890	JAASUI000000000	MT124497
L. farberi	FSL-L7-0072	TX	29.55308	-98.7545	2.95	36.85	14	539249	343	SRR11343947	JAARZO00000000	
L. farberi	FSL-L7-0083	TX	29.5391	-98.75099	2.94	36.91	15	1483358	242	SRR11343913	JAARZL000000000	
L. farberi	FSL-L7-0091^{T}	TX	31.03675	-97.5287	3.02	36.75	61	168881	95	SRR11344176	JAARZJ000000000	MT117899
L. farberi	FSL L7-1693	FL	29.8537	-81.87718	2.86	36.84	16	1512479	94	SRR11344027	JAARPJ000000000	
L. farberi	FSL L7-1699	FL	29.84415	-81.87673	2.99	36.82	86	1162269	154	SRR11344025	JAARPH000000000	
L. immobilis	FSL-L7-1485	WY	44.0632703	-104.6894801	2.91	35.94	60	155385	114	SRR11343882	JAASUF000000000	
L. immobilis	FSL-L7-1509	SD	43.9100485	-102.6949291	3.04	35.94	62	183435	101	SRR11343875	JAASUD000000000	
L. immobilis	FSL-L7-1510	SD	43.9100485	-102.6949291	2.94	35.93	46	214661	58	SRR11343874	JAASUC000000000	
L. immobilis	FSL-L7-1515	SD	43.8953882	-102.6854116	3.07	35.94	52	177674	49	SRR11344254	JAASUB000000000	
L. immobilis	FSL-L7-1517	WY	43.890325	-107.6895718	2.98	35.78	37	227776	49	SRR11344253	JAASUA000000000	
L. immobilis	FSL-L7-1519^{T}	WY	43.890325	-107.6895718	3.07	35.92	46	156931	47	SRR11344252	JAASTZ000000000	MT117845
L. immobilis	FSL-L7-1554	MT	46.0519691	-105.5742674	2.92	35.92	44	144674	39	SRR11344243	JAASTW000000000	
L. immobilis	FSL-L7-1558	MT	46.0519691	-105.5742674	2.94	35.92	61	108742	33	SRR11344242	JAASTV000000000	
L. immobilis	FSL-L7-1578	SD	44.7082988	-103.7171173	3.02	35.96	53	138184	46	SRR11344237	JAASTU000000000	
L. portnoyi	FSL L7-1582^{T}	SD	44.7082988	-103.71712	3.16	41.9	62	125185	55	SRR11344233	JAASTT000000000	MT117898
L. rustica§	FSL W9-0585^{T}	NY	42.422845	-76.832515	3.49	42.25	65	171825	123	SRR11780234	JABJVM000000000	MT117896

*The Sequence Read Archive (SRA) database

†The GenBank Genome database for the assembled draft genomes

#The GenBank database for prokaryotic 16S rRNA. 16SrRNA sequences were extracted from the draft genomes of the type strains only

§*L. rustica* is the only isolate obtained from agricultural water, all other isolates originated from soil

Table S2. Bacterial numbers of the novel *Listeria* spp. and two subspecies following the temperature growth experiments

**Novel Species**	**Isolate**	**Log CFU/mL at each incubation time and temperature**†									
		4˚C		22˚C		30˚C		37˚C		41˚C	
		10 d	14 d	24 h	48 h	24 h	48 h	24 h	48 h	24 h	48 h
L. cossartiae subsp. *cossartiae*	FSL L7-1447ᵀ	1.44	2.93	4.03	6.84	6.96	6.34	6.72	5.95	6.72	5.77
L. cossartiae subsp. *cossartiae*	FSL L7-0253	1.68	2.92	5.05	7.26	7.21	6.16	7.14	6.58	7.35	6.42
L. cossartiae subsp. *cayugensis*	FSL L7-0993ᵀ	1.68	3.21	5.17	7.58	7.58	6.52	7.01	6.67	7.15	6.35
L. farberi	FSL L7-0091ᵀ	1.78	2.93	4.60	6.78	6.72	6.60	6.77	6.16	6.74	5.82
L. immobilis	FSL L7-1517	1.29	1.85	4.86	7.31	7.33	7.05	7.17	6.56	5.49	6.70
L. immobilis	FSL L7-1519ᵀ	1.31	2.16	3.85	6.86	6.71	6.64	6.81	6.27	5.20	5.96
L. immobilis	FSL L7-1554	1.35	1.89	3.44	6.93	7.08	7.15	7.30	6.31	4.09	6.72
L. portnoyi	FSL L7-1582ᵀ	3.5	5.18	6.44	5.50	6.50	5.29	-1.09*	No Growth	-0.78*	No Growth
L. rustica	FSL W9-0585ᵀ	3.11	4.79	4.91	6.20	6.44	6.12	1.34	1.73	-0.76*	No Growth
L. monocytogenes	10403S‡	1.21	2.54	4.81	7.61	7.54	6.74	7.34	6.62	7.34	6.44

†The average CFU/mL of two biological replicates from each time and temperature combination after accounting for the starting inoculum. Each biological replicate is the average of two technical replicates. All isolate-temperature combinations that yielded “No Growth” were verified with a third biological replicate.

*Negative bacterial numbers reflect a reduction from the starting inoculum.

‡*L. monocytogenes* 10403S was used as a control sample.

Table S3. Biochemical results from API 50CH not reported in Table 3

	Novel Species				
	sensu stricto			*sensu lato*	
	**Lcs**	**Lfr**	**Lim**	**Lpo**	**Lru**
Erythritol	-	-	-	-	-
D-Arabinose	-	-	-	-	-
L-Xylose	-	-	-	-	-
D-Adonitol	-	-		-	-
Methyl-α-D-Xylopyranoside	-	-	-	-	-
D-Fructose	+	+	+	+	+
D-Mannose	+	+	+	+	+
Dulcitol	-	-	-	-	-
D-Sorbitol	-	-	-	-	-
N-Acetylglucosamine	+	+	+	+	+
Amygladin	+	+	+	+	+
Arbutin	+	+	+	+	+
Salicin	+	+	+	+	+
D-Cellobiose	+	+	+	+	+
D-Trehalose	V	-	(+)	+	+
D-Rafinose	-	-	-	-	-
Amidon	-	-	-	-	-
Glycogen	-	-	-	-	-
Xylitol	+	+	-	-	-
Gentiobiose	+	+	+	+	+
D-Fucose	-	-	-	-	-
L-Fucose	-	-	-	-	-
L-Arabitol	-	-	-	-	-
Potassium Gluconate	-	-	-	-	-
Potassium 2-Ketogluconate	-	-	-	-	-
Potassium 5-Ketogluconate	-	-	-	-	-

+ positive; (+) weak positive; V variable between strains

Table S4. *Listeria* spp. flagellar motility genes from the cgMSLT1748 scheme from the Institut Pasteur open access BIGSDB-*Lm* platform

Locus	Full name/product
lmo0676	flagellar biosynthesis protein FliP
lmo0677	flagellar biosynthesis protein FliQ
lmo0678	flagellar biosynthesis protein FliR
lmo0680	flagellar biosynthesis protein FlhA
lmo0681	flagellar biosynthesis regulator FlhF
lmo0682	flagellar basal body rod protein FlgG
lmo0685	flagellar motor protein MotA
lmo0686	flagellar motor rotation MotB
lmo0690	flagellin
lmo0693	flagellar motor switch protein FliY
lmo0696	flagellar basal body rod modification protein
lmo0697	flagellar hook protein FlgE
lmo0698	flagellar motor switch protein
lmo0699	flagellar motor switch protein FliM
lmo0700	flagellar motor switch protein FliY
lmo0705	flagellar hook-associated protein FlgK
lmo0706	flagellar hook-associated protein FlgL
lmo0707	flagellar capping protein FliD
lmo0708	flagellar protein
lmo0710	flagellar basal-body rod protein FlgB
lmo0711	flagellar basal body rod protein FlgC
lmo0712	flagellar hook-basal body protein FliE
lmo0713	flagellar MS-ring protein FliF
lmo0714	flagellar motor switch protein FliG
lmo0715	flagellar assembly protein H
lmo0716	flagellum-specific ATP synthase

Table S5. Results from the novel species draft genome analyses for antibiotic resistance genes, prophages and plasmids

Proposed Name	Strain	AMR Genes[†]			Prophage[*]			Plasmid[‡]
		FosX	*lin*	*Listeria monocytogenes mprF*	Intact (>90)	Questionable (70-90)	Incomplete (<70)	
L. cossartiae subsp. *cayugensis*	FSL L7-0123	+	+	+	-	-	3	1
L. cossartiae subsp. *cayugensis*	FSL L7-0993[T]	+	+	+	1	-	1	1
L. cossartiae subsp. *cossartiae*	FSL L7-0229	+	+	-	-	1	1	-
L. cossartiae subsp. *cossartiae*	FSL L7-0233	+	+	+	-	-	2	1
L. cossartiae subsp. *cossartiae*	FSL L7-0253	+	+	-	-	-	2	1
L. cossartiae subsp. *cossartiae*	FSL L7-0478	+	+	+	-	-	1	-
L. cossartiae subsp. *cossartiae*	FSL-L7-1425	+	+	+	-	-	2	-
L. cossartiae subsp. *cossartiae*	FSL-L7-1426	+	+	-	-	-	1	2
L. cossartiae subsp. *cossartiae*	FSL-L7-1434	+	-	+	-	1	2	-
L. cossartiae subsp. *cossartiae*	FSL-L7-1435	+	+	+	-	-	2	-
L. cossartiae subsp. *cossartiae*	FSL-L7-1447[T]	+	+	-	-	-	2	-
L. farberi	FSL-L7-0072	+	-	-	1	-	1	1
L. farberi	FSL-L7-0083	+	-	-	-	-	3	-
L. farberi	FSL-L7-0091[T]	+	-	-	-	-	1	-
L. farberi	FSL L7-1693	+	-	-	-	-	1	-
L. farberi	FSL L7-1699	+	-	-	1	1	1	-
L. immobilis	FSL-L7-1485	+	-	-	1	-	1	2
L. immobilis	FSL-L7-1509	+	-	-	1	1	2	3
L. immobilis	FSL-L7-1510	+	-	-	-	1	2	2
L. immobilis	FSL-L7-1515	+	-	-	3	-	3	2
L. immobilis	FSL-L7-1517	+	-	-	1	-	2	2
L. immobilis	FSL-L7-1519[T]	+	-	-	3	-	3	2
L. immobilis	FSL-L7-1554	+	-	-	1	-	2	2
L. immobilis	FSL-L7-1558	+	-	-	1	-	2	3
L. immobilis	FSL-L7-1578	+	-	-	2	-	5	2
L. portnoyi	FSL L7-1582[T]	-	-	-	1	-	4	-
L. rustica	FSL W9-0585[T]	-	-	-	-	-	-	-

[†]AMR gene hits from Resistance Gene Identifier (RGI; https://card.mcmaster.ca/analyze/rgi) analyses.

[*] The number of intact prophages detected using PHASTER (https://phaster.ca/). Completeness scores (intact, questionable, incomplete) are based on the proportion of phage genes present in the draft genome.

[‡] The number of putative plasmid sequences detected using Platon (https://github.com/oschwengers/platon)

Table S6: *In silico* DNA-DNA Hybridization (DDH) values between the *L. cossartiae* subspecies isolates

	L. cossartiae subsp. *cayugensis* L7-0123			*L. cossartiae* subsp. *cayugensis* L7-0993[T]		
	isDDH	CI[†]	Probability DDH >70% and >79%[‡]	isDDH	CI[†]	Probability DDH >70% and >79%[‡]
L. cossartiae subsp. *cossartiae* L7-0229	58.6%	55.8-61.4%	47.58% and 11.30%	60.9%	58.8-63.7%	55.25% and 14.14%
L. cossartiae subsp. *cossartiae* L7-0233	58.9%	56.1-61.7%	48.77% and 11.70%	60.1%	57.2-62.9%	52.63% and 13.76%
L. cossartiae subsp. *cossartiae* L7-0253	59.0%	56.1-61.7%	48.82% and 11.72%	60.6%	57.7-63.4%	54.32% and 13.10%
L. cossartiae subsp. *cossartiae* L7-0478	58.6%	55.8-61.3%	47.46% and 11.25%	61.9%	59.0-64.7%	58.38% and 15.48%
L. cossartiae subsp. *cossartiae* L7-1425	59.1%	56.3-61.8%	49.23% and 11.86%	61.6%	58.7-64.4%	57.55% and 15.11%
L. cossartiae subsp. *cossartiae* L7-1426	58.8%	56.0-61.5%	48.18% and 11.50%	61.9%	59.0-64.7%	58.42% and 15.50%
L. cossartiae subsp. *cossartiae* L7-1434	59.0%	56.2-61.8%	49.05% and 11.80%	61.5%	58.7-64.3%	57.33% and 15.01%
L. cossartiae subsp. *cossartiae* L7-1435	59.0%	52.6-61.8%	49.05% and 11.80%	61.8%	58.9-64.6%	58.04% and 15.33%
L. cossartiae subsp. *cossartiae* L7-1447[T]	59.1%	56.3-61.9%	49.35% and 11.90%	61.5%	58.6-64.3%	57.09% and 14.91%

[†] Confidence interval from the generalized linear model (GLM) used to infer isDDH (44).

[‡] The probability from a logistic regression model that DDH is >70% (i.e., same species) and >79% (i.e., same subspecies) (44).

Fig. S1

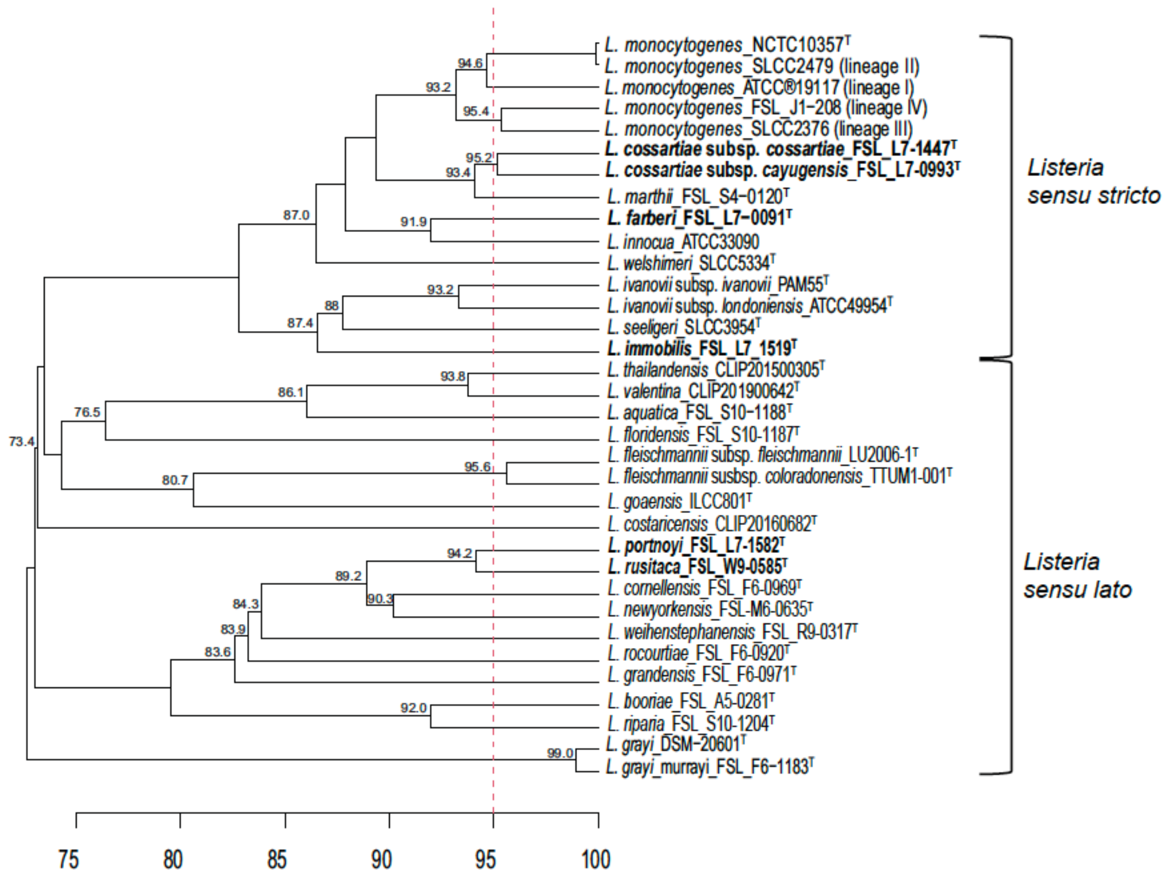

L. monocytogenes_NCTC10357T
L. monocytogenes_SLCC2479 (lineage II)
L. monocytogenes_ATCC®19117 (lineage I)
L. monocytogenes_FSL_J1-208 (lineage IV)
L. monocytogenes_SLCC2376 (lineage III)
L. cossartiae subsp. cossartiae_FSL_L7-1447T
L. cossartiae subsp. cayugensis_FSL_L7-0993T
L. marthii_FSL_S4-0120T
L. farberi_FSL_L7-0091T
L. innocua_ATCC33090
L. welshimeri_SLCC5334T
L. ivanovii subsp. ivanovii_PAM55T
L. ivanovii subsp. londoniensis_ATCC49954T
L. seeligeri_SLCC3954T
L. immobilis_FSL_L7_1519T
L. thailandensis_CLIP201500305T
L. valentina_CLIP201900642T
L. aquatica_FSL_S10-1188T
L. floridensis_FSL_S10-1187T
L. fleischmannii subsp. fleischmannii_LU2006-1T
L. fleischmannii susbsp. coloradonensis_TTUM1-001T
L. goaensis_ILCC801T
L. costaricensis_CLIP20160682T
L. portnoyi_FSL_L7-1582T
L. rusitaca_FSL_W9-0585T
L. cornellensis_FSL_F6-0969T
L. newyorkensis_FSL-M6-0635T
L. weihenstephanensis_FSL_R9-0317T
L. rocourtiae_FSL_F6-0920T
L. grandensis_FSL_F6-0971T
L. booriae_FSL_A5-0281T
L. riparia_FSL_S10-1204T
L. grayi_DSM-20601T
L. grayi_murrayi_FSL_F6-1183T
Listeria sensu stricto
Listeria sensu lato
94.6
93.2
95.4
95.2
93.4
87.0
91.9
93.2
88
87.4
93.8
86.1
76.5
73.4
95.6
80.7
94.2
89.2
90.3
84.3
83.9
83.6
92.0
99.0
75
80
85
90
95
100
